# Antioxidant activity of linalool in patients with carpal tunnel syndrome

**DOI:** 10.1186/s12883-016-0541-3

**Published:** 2016-02-02

**Authors:** Geun-Hye Seol, Purum Kang, Hui Su Lee, Geun Hee Seol

**Affiliations:** Department of Basic Nursing Science, School of Nursing, Korea University, 145 Anam-ro, Seongbuk-gu, Seoul, 02841 Republic of Korea

**Keywords:** Carpal tunnel syndrome, Linalool, Antioxidative activity

## Abstract

**Background:**

Carpal tunnel syndrome (CTS) is a common peripheral neuropathy and ischemic-reperfusion injury. Oxidative stress is considered a major cause of CTS. Linalool, a component of essential oils, has antioxidant activity. This study was designed to determine the effects of linalool inhalation on oxidative stress in patients with CTS.

**Methods:**

This double-blind, placebo-controlled study assessed the effects of linalool inhalation on oxidative stress in patients with CTS. Thirty-seven subjects, with and without CTS, were randomized to inhalation of 1 % linalool or carrier oil. 1,1-Diphenyl-2-picrylhydrazyl (DPPH) radical scavenging activity, systolic blood pressure (sBP), diastolic blood pressure (dBP) and pulse rate were analyzed.

**Results:**

DPPH inhibition was significantly higher in both experimental groups than in their respective controls. Moreover inhalation of linalool reduced sBP, dBP and pulse rate in the CTS group, and pulse rate in the non-CTS group. However, there were no significant differences among the study groups in nitrite levels, sBP, dBP and pulse rate.

**Conclusions:**

Inhalation of linalool increases antioxidative activity and reduces blood pressure and pulse rate in patients with CTS.

## Background

Carpal tunnel syndrome (CTS) is one of the most commonly observed neuropathies. CTS is typically caused by compression of the median nerve at the wrist due to elevated tunnel pressure [1]. Such compression may be associated with repeated trauma caused by work, pregnancy or type 2 diabetes, but in most patients CTS is idiopathic [2]. Ischemia-reperfusion injury in subsynovial connective tissue may also cause CTS [3]. Reactive oxygen species (ROS) derived from oxidative stress contribute to tissue injury, and may therefore cause and exacerbate CTS [4]. Oxidative stress in subsynovial connective tissue has been associated with CTS. Moreover, the subjective symptoms of CTS were found to be triggered by oxidative stress and activation of proinflammatory cytokines [5]. In addition, oxidative stress level and antioxidant activity were found to be altered in patients with CTS [6].

Patients with CTS experience pain, numbness and tingling and functional deficits in the wrist and hand that affect their daily life [1]. Severe CTS may result in weakness in the affected hand, frustration or inability related to motor deficits [7]. Non-surgical interventions in patients with CTS include analgesics, splinting and steroid injections, although surgery may be required in patients with intractable or severe diseases. Injection of steroids results in significant symptom relief, but it is unfit for long-term therapy [8]. Splinting the wrist reduces pressure on the median nerve, but its effect on long-term symptoms is unclear [9]. Due to the limitations of these conservative treatments, additional interventions are required for the symptomatic relief of patients with CTS.

Linalool, a monoterpene alcohol, is a component of many natural aromatic plants. Linalool has been found to have biological activities, including analgesic, anti-inflammatory and antioxidant effects. The anti-inflammatory effects of linalool in lung cells have been associated with the modulation of pro-inflammatory cytokines and antioxidant enzymes. In particular, linalool reduced the levels of nuclear factor-erythroid 2, a regulator of antioxidant stress [10]. In addition, linalool was found to be effective as an antioxidant in guinea pig brains injected with H_2_O_2_ [11], one of the major reagents used in antioxidant studies. Also, in male Wistar rats, linalool decreased oxidative stress by modulating malondialdehyde, a marker for lipid peroxidation and increased glutathione content [12]. Moreover, linalool had an analgesic effect in an animal model of acute pain induced by paclitaxel, a widely used chemotherapy agent [13]. The antioxidant properties of linalool suggested it may have beneficial effects in patients with CTS. This study therefore assessed the effects of linalool inhalation on antioxidant activity in patients with CTS.

## Methods

### Study design and patient population

The effects of linalool on antioxidative activity in patients with carpal-tunnel syndrome were assessed using a pretest-posttest control group study design. Healthy adults with no underlying disease (non-CTS group) were recruited. In addition, patients with CTS diagnosed by electrodiagnostic tests (CTS group) who were scheduled for outpatient surgery at the Center for Plastic and Reconstructive Surgery in Seoul, Korea, were recruited. Each subject was randomly assigned to inhale linalool or carrier oil. Adequate sample size was determined using the G-Power program. Based on a statistical power of 0.50, an effect size of 0.40, and a significance level of 0.05, the minimum number of total patients required to compare differences between the experimental groups was estimated to be 41 patients.

After the study protocol was approved by the Ethical Review Committee of the Korea University Medical Center’s approval (Code: ED12257), subjects were recruited for 18 months, starting in June 2013. In addition to providing written informed consent for study participation, all subjects were required to have no history of psychiatric illness, no disturbances of olfactory acuity, no experience with aromatherapy and to be free of allergies to linalool. Patients with CTS were diagnosed by a registered physician and had not undergone surgery. After exclusion of participants who dropped out of the study, 37 individuals completed the study (14/37 non-CTS group with linalool; 6/37 non-CTS group with carrier; 11/37 CTS group with linalool; and 6/37 CTS group with carrier).

### Intervention

Linalool was purchased from Sigma (St. Louis, MO, USA) and almond oil was obtained from Aromarant Co. Ltd. (Rottingen, Germany). Linalool was dissolved in almond oil at a concentration of 1 %. A 0.5 mL aliquot of 1 % linalool in almond oil (or almond oil alone) was placed onto a gauze pad (3 × 2 cm^2^), and the pad was positioned five centimeters from the nose of each subject for 10 min, with the subject in a sitting position after deep breathing. Before the intervention, general characteristics, blood pressure and heart rates were measured. After the intervention, blood samples were collected and blood pressure and heart rates were measured.

### Blood pressure and pulse rate measurements

Blood pressure and heart rate as indicators of the response of the autonomic nervous system were measured before and after linalool or carrier oil inhalation. Blood pressure was measured in the right brachial artery after a 10-min rest in a sitting position. Heart rate was measured at the radial artery for 1 min.

### Assay of 1,1-Diphenyl-2-picrylhydrazyl (DPPH) radical scavenging activity

Plasma concentrations of the radical scavenger 1,1-diphenyl-2-picrylhydrazyl (DPPH) were measured. Blood samples (3 mL) were obtained from each participant after inhalation and centrifuged at 3500 rpm for 10 min at 4 °C to separate the plasma. The samples were stored at −80 °C until assayed. Proteins were removed by mixing plasma and acetonitrile at a 1:1 ratio, incubating the samples at room temperature for 2 min, and centrifuging the samples at 9500 × g for 10 min at 4 °C. The resultant supernatants were diluted five-fold with ethanol. DPPH in anhydrous ethanol was mixed 1:1 with Tris HCL and added to 96 well plates, to which were added plasma supernatants. Ascorbic acid was used as the standard antioxidant. The 96 well plates were incubated in the dark at 37 °C for 30 min, and the absorbances of the solutions were measured spectrophotometrically at 517 nM using a microplate reader. The percentage of scavenging activity was compared with that obtained in the sample of ascorbic acid.

DPPH free radical scavenging activity (%) was defined as (1-sample absorbance/control absorbance)*100.

### Statistical analysis

All statistical analyses were performed using SPSS software (version 12.0). Participants’ characteristics at baseline were explored using Fisher’s exact test. A Shapiro-Wilk test was applied to evaluate data normality, and differences among groups were tested by the Kruskal-Wallis test or one-way analysis of variance (ANOVA). Within group differences in scores before and after interventions were compared using the Wilcoxon’s rank-sum test. All data were reported as mean ± standard deviation (SD), with *P*-values < .05 considered statistically significant.

## Results

### Characteristics of study population

Thirty seven women were screened for eligibility and participated in the study. Mean subject age was 59.9 years; the general characteristics of the participants are shown in Table 1. At baseline, the demographic and disease-related characteristics of the groups were similar, including body mass index (BMI) and treatments with antihypertensive and analgesic agents (Table 2).

**Table 1:** General characteristics of the subjects

Characteristics	non-CTS Control *n* (%)	non-CTS Linalool *n* (%)	CTS Control *n* (%)	CTS Linalool *n* (%)	*P*-value^a^
Age (years)					0.570
41–50	1 (16.7)	1 (7.1)	1 (16.7)	1 (9.1)	
51–60	1 (16.7)	5 (35.7)	2 (33.3)	6 (54.5)	
61–70	4 (66.7)	8 (57.1)	2 (33.3)	3 (27.3)	
≥ 71	0 (0)	0 (0)	1 (16.7)	1 (9.1)	
Gender					
Male	0 (0)	0 (0)	0 (0)	0 (0)	
Female	6 (100)	14 (100)	6 (100)	11 (100)	
Marriage					0.324
Yes	6 (100)	14 (100)	5 (83.3)	11 (100)	
No	0 (100)	0 (0)	1 (16.2)	0 (0)	
Occupation					0.138
Housewife	6 (100)	9 (64.3)	4 (66.7)	8 (72.7)	
Other	0 (0)	5 (35.7)	2 (33.4)	3 (27)	
BMI (kg/m2)					0.465
< 18.5	1 (16.7)	6 (42.9)	1 (16.7)	5 (45.5)	
18.5 ~ 23.0	4 (66.7)	8 (57.1)	5 (83.3)	5 (45.5)	
> 23.0	1 (16.7)	0 (0)	0 (0)	1 (9.1)	
Menopause					0.053
Yes	5 (83.3)	14 (100)	5 (83.3)	7 (63.6)	
No	1 (16.7)	0 (0)	1 (16.7)	4 (36.4)	
Analgesics					
Yes	0	0 (0)	0 (0)	0 (0)	
No	6 (100)	14 (100)	6 (100)	11 (100)	
Antihypertensive drug					0.250
Yes	3 (50)	3 (21.4)	0 (0)	2 (18.2)	
No	3 (30)	11 (78.6)	6 (100)	9 (81.8)	

^a^By Fisher’s exact test. CTS, carpal tunnel syndrome

**Table 2:** Homogeneity test for measurement variables among four groups at pretest

Variables	non-CTS Control	non-CTS Linalool	CTS Control	CTS Linalool	*P*-value^a^
sBP	125.00 ± 10.49	120.00 ± 9.61	130.00 ± 8.94	124.55 ± 15.08	0.267
dBP	78.33 ± 7.53	74.29 ± 7.56	79.67 ± 5.72	79.09 ± 5.39	0.221
Pulse rate	71.50 ± 8.71	75.00 ± 8.38	86.67 ± 12.75	70.36 ± 10.61	0.065

Data presented as mean ± standard deviation

*T-VAS* tingling visual analogue scale, *sBP* systolic blood pressure, *dBP* diastolic blood pressure, *CTS* carpal tunnel syndrome

^a^Analyzed using Kruskal-Wallis test

### Effects of linalool on the DPPH radical scavenging activity

**Fig. 1:**
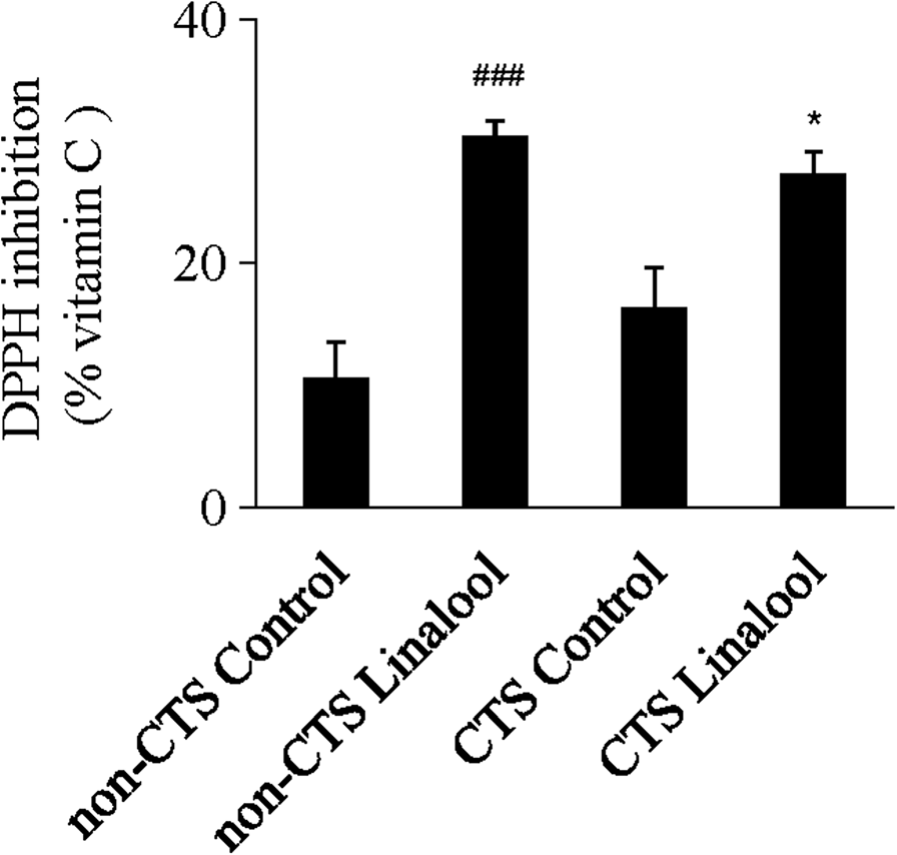
Effects of linalool inhalation on DPPH among four groups after treatment. Results presented as mean ± standard error of the mean, with differences compared by one-way ANOVA followed by Tukey’s post hoc test. *P < 0.05 compared with CTS control group. ^###^ *P* < 0.001 compared with non-CTS control group. CTS, carpal tunnel syndrome

Mean DPPH radical-scavenging activities in plasma samples from the non-CTS control, non-CTS linalool, CTS control and CTS linalool groups were 10.40 ± 3.08 %, 30.38 ± 1.32 %, 16.32 ± 3.39 %, and 27.36 ± 1.82 %, respectively (Fig. 1). Differences among the 4 groups were analyzed by one-way ANOVA followed by Tukey’s post hoc test. DPPH radical scavenging activity was higher in the CTS linalool groups compared with their respective CTS control groups (*P* = 0.031) and in non-CTS linalool groups compared with their respective control groups (*P* < 0.001).

### Effects of linalool on blood pressure and heart rate

Following inhalation of linalool, both systolic (*P* = 0.004) and diastolic (*P* = 0.025) blood pressure significantly decreased from baseline in subjects with CTS, but there were no differences among the four groups (Table 3). Inhalation of linalool significantly reduced heart rate compared with baseline in both the non-CTS linalool (*P* = 0.004) and CTS linalool (*P* = 0.017) groups, although there were no significant differences among the four groups after inhalation.

**Table 3:** Difference in dependent variables between four groups after treatment

Variables	non-CTS Control	non-CTS Linalool	CTS Control	CTS Linalool	*P*-value^a^
sBP					
Pre-test	125.00 ± 10.49	120.00 ± 9.61	130.00 ± 8.94	124.55 ± 15.08	
Post-test	121.67 ± 9.83	117.14 ± 10.69	128.33 ± 9.83	114.55 ± 16.95	
Difference	3.33 ± 13.66	2.86 ± 10.69	1.67 ± 17.22	10.00 ± 8.94	0.237
*P*-value^b^	0.576	0.336	0.822	0.004	
dBP					
Pre-test	78.33 ± 7.53	74.29 ± 7.56	79.67 ± 5.72	79.09 ± 5.39	
Post-test	81.67 ± 9.83	72.14 ± 8.93	78.33 ± 9.83	73.64 ± 9.24	
Difference	−3.33 ± 5.16	2.14 ± 8.02	1.33 ± 11.08	5.45 ± 6.88	0.192
*P*-value^b^	0.175	0.336	0.780	0.025	
Pulse rate					
Pre-test	71.50 ± 8.71	75.00 ± 8.38	86.67 ± 12.75	70.36 ± 10.61	
Post-test	69.83 ± 4.83	71.36 ± 7.98	85.33 ± 11.64	65.55 ± 8.80	
Difference	1.67 ± 6.22	3.64 ± 0.97	1.33 ± 5.32	4.82 ± 5.60	0.435
*P*-value^b^	0.540	0.004	0.566	0.017	

Data presented as mean ± standard deviation

*T-VAS* tingling visual analogue scale, *sBP* systolic blood pressure, *dBP* diastolic blood pressure, *CTS* carpal tunnel syndrome

^a^Differences among groups analyzed using the Kruskal-Wallis test

^b^Within group differences analyzed using Wilcoxon’s rank-sum test

## Discussion

The principal aim of this study was to evaluate the efficacy of linalool as an antioxidant in patients with CTS. We hypothesized that linalool would enhance oxidative defenses in healthy adults. Indeed, we found that inhalation of linalool by subjects with no underlying disease significantly improved antioxidant activity, in agreement with previous results showing that linalool decreased tissue injury evoked by oxidative stress in rats [14].

Oxidative stress is characterized by an imbalance between generation and removal of ROS, and has been shown to contribute to the pathogenesis of various diseases throughout the body [15]. The cumulative effect of ROS from ischemia reperfusion on the flexor tenosynovium and subsynovial connective tissue has been found to lead to CTS [3]. Similarly, the pro-inflammatory cytokine activator nuclear factor κB (NF-κB) and transforming growth factor were found to be elevated in patients with CTS. Moreover, the level of oxidative stress correlated with the degree of subjective symptoms [5], with both antioxidant activity and oxidative stress altered in patients with CTS [6]. We therefore evaluated the effects of linalool on DPPH radical scavenging activity in subjects without underlying disease and in individuals with CTS. We observed significant relationships between linalool and antioxidant level, not only in subjects without underlying disease but in patients with CTS. These results therefore indicated that inhalation of linalool enhanced antioxidant activity, regardless of the presence of increased oxidative stress. These findings, along with a report showing that linalool effectively decreased NF-κB in diabetic rats [16], suggest that linalool may be used to treat symptoms in patients with CTS.

Linalool is a natural compound with numerous pharmacological activities, acting not only as an antioxidant but as a cardiovascular modulator, analgesic and antianxiety agent. Transdermal absorption of linalool reduced blood pressure in healthy subjects compared with a control group, with no side effects [17]. Moreover, linalool was found to reduce heart rate and stably alter mood status in healthy volunteers [18]. In rats and mice, linalool decreased blood pressure by modulating blood vessels [19, 20], and was reported to have sedative or anxiolytic-like effects in mice [21]. Similarly, we confirmed that linalool decreased heart rate and blood pressure in subjects with CTS.

CTS is a common chronic condition. Subjects usually experience sensory changes, including tingling and numbness. Symptoms may continue or worsen, with subjects experiencing sharp pain and weakness, and eventually complaining of reduced ability or frustration related to motor deficits [7]. Therefore, linalool may relieve symptoms and enhance emotional stability in patients with CTS.

Current interventions for CTS result in partial symptom relief and are not effective in all patients. Therefore, there is a need to develop additional treatments. Inhalation therapy is non-invasive and simple, suggesting that it may be a new option for patients with CTS.

## Conclusion

Our results indicate that inhalation of linalool increased antioxidative activity not only in healthy adults but also in CTS patients, and reduced blood pressure and pulse rate in CTS patients. These findings suggest that linalool may be a useful additional intervention in these patients.

## Abbreviations

ANOVA: Analysis of variance
BMI: Body mass index
CTS: Carpal tunnel syndrome
dBP: Diastolic blood pressure
DPPH: 1,1-Diphenyl-2-picrylhydrazyl
NF-κB: Nuclear factor κB
ROS: Reactive oxygen species
sBP: Systolic blood pressure
SD: Standard deviation
